# Exogenous recombinant Hsp70 attenuates sevoflurane anesthesia‐induced cognitive dysfunction in aged mice

**DOI:** 10.1002/brb3.2861

**Published:** 2022-12-27

**Authors:** Yongxiang Xie, Jianzhong Huang, Yijia Chen

**Affiliations:** ^1^ Department of Anesthesiology Longyan People Hospital of Fujian Longyan China; ^2^ Department of Anesthesiology Zhangzhou Affiliated Hospital of Fujian Medical University Zhangzhou China

**Keywords:** aging, cognitive function, neuroinflammation, recombinant Hsp70, sevoflurane

## Abstract

**Background:**

Postoperative cognitive dysfunction (POCD) is a severe postoperative neurological sequela in elderly patients, and there is currently no standard treatment for POCD. In this study, whether recombinant human heat shock protein 70 (rHsp70) could alleviate sevoflurane‐induced cognitive impairment in aged mice is investigated.

**Methods:**

To determine the prophylactic effect of rHsp70 in sevoflurane‐induced cognitive dysfunction, aged mice were pretreated with different concentrations of rHsp70 (29.4, 58.8, and 117.6 μg/kg; intranasal injected; N = 12) every day for 1 week; then, 3% sevoflurane was utilized to anesthetize the aged mice. Cognitive function, neurotoxicity, and serum and hippocampal Hsp70 levels in aged mice undergoing sevoflurane anesthesia were assessed by the Morris water maze test and enzyme‐linked immunosorbent assay. The effects of rHsp70 on inflammatory response were assessed by proinflammatory cytokine production and nuclear factor‐κB (NF‐κB) activation assays.

**Results:**

We found that aged mice exposed to sevoflurane showed reduced learning and memory ability and reduced Hsp70 expression, which were both restored by rHsp70 pretreatment. RHsp70 also reversed sevoflurane‐induced up‐regulated Bax and Bcl‐2 expression and interleukin‐1, IL‐6, and monocyte chemoattractant protein‐1 overproduction. Finally, rHsp70 pretreatment suppressed sevoflurane‐induced NF‐κB activation. Our study indicated that rHsp70 was sufficient to suppress sevoflurane‐induced cognitive decline and neurotoxicity.

**Conclusion:**

Our important finding warrants further study on the clinical application of rHsp70 in elderly patients undergoing anesthesia.

## Graphical abstract

1. rHsp70 pretreatment suppressed sevoflurane induced NF‐?B activation.

2>. rHsp70 is sufficient to suppress sevoflurane induced cognitive decline and neurotoxicity.

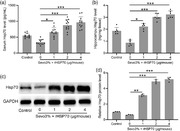

## 1 INTRODUCTION

Postoperative cognitive decline (POCD) is a major complication among aging patients undergoing anesthesia and surgery (Terrando et al., 2011). About a quarter of elderly patients greater than 60 years old have POCD 1 week after surgery, and POCD will persistently affect 10% of these patients (Newfield, 2009). Sevoflurane is a commonly used inhalable anesthetic that has been shown to induce cognitive impairment and prolong neurocognitive recovery among elderly patients (Konishi et al., 2018; Zhang et al., 2018). In young mice, sevoflurane could induce spatial memory and long‐term memory impairment indicated by Morris water maze (Wang et al., 2019). While in senile mice, in addition to spatial memory and long‐term memory impairment, long‐term depression could also be induced by sevoflurane anesthesia (X. Yu et al., 2019).

Neuroinflammation is associated with the pathogenesis of sevoflurane‐induced cognitive dysfunction (Lv et al., 2020). Mice exposed to sevoflurane demonstrate increased proinflammatory cytokines production and TLR4‐NF‐κB signaling activation, and TLR4 deletion could protect aged mice from sevoflurane‐induced cognitive decline along with suppressed cytokine production (Fei et al., 2020). Consistently, another study shows that rapamycin alleviates cognitive deficits by modulating the TLR4‐NF‐κB signaling, leading to activated autophagy, suppressed apoptosis, and reduced damage to the brain tissue (Y. Li, Liu, et al., 2019). We thus speculate that proteins that are involved in modulating inflammatory responses and neuroprotection may be an ideal target for developing therapeutic strategies in the treatment of sevoflurane‐induced neurotoxicity.

The heat shock proteins (Hsps) are a family of chaperones, which can protect neurons from toxicity and protein aggregation in Parkinson's disease, Alzheimer's disease, polyglutamine diseases, and amyotrophic lateral sclerosis; protect cells from apoptosis in Parkinson's disease; and protect cells from cerebral ischemic injury‐induced inflammation (Hu et al., 2022; Turturici et al., 2011). Hsp70 is a member of the Hsp family that exerts a neuroprotective role in aging animal models and other disease scenarios (Mohammadi et al., 2018). Chronic administration of exogenous recombinant Hsp70 (rHsp70) leads to increased lifespan and improved cognitive function in aging mice, including learning and memory ability (Bobkova et al., 2015). A previous study also shows that the intranasal administration of rHsp70 in Alzheimer's disease mouse models alleviates cognitive abnormalities, decreases pyknosis, karyolysis, cytolysis formation in neurons, and reduces amyloid‐beta plaque formation, and improves spatial memory (Bobkova et al., 2014). Hsp70 has also been shown to be implicated in the pathogenesis of sevoflurane‐induced cognitive impairment. When treated human neuroglioma cells with 17AAG, an Hsp90 inhibitor, Hsp70 is activated and suppresses neurotoxicity induced by sevoflurane. On the other hand, Hsp70 knock‐down effectively blocks the neuroprotective effect of 17AAG in response to sevoflurane‐induced neurotoxicity (Liu et al., 2020).

Multiple molecular mechanisms have been reported by which Hsp70 exerts its neuroprotective effects, including cell survival and apoptosis regulations, toxic protein aggregation, and oxidative stress relief. One particular interest to us in this study is the role of Hsp70 in suppressing inflammatory signaling. Hsp70 has been shown to exert an anti‐inflammatory effect and suppress α‐synuclein‐induced neuroinflammation by inhibiting pro‐inflammatory cytokine production and NF‐κB signaling (W. W. Yu et al., 2018).

We thus hypothesize that rHsp70 might alleviate sevoflurane‐induced cognitive dysfunction in aged mice. In this study, we test this hypothesis by pretreating the mice with exogenous rHsp70 prior to sevoflurane exposure in aged mice and investigate the potential cellular and molecular mechanism underlying rHsp70‐mediated neuroprotective effects.

## 2 MATERIALS AND METHODS

### 2.1 Mice and drug treatments

RHsp70 was prepared as described previously (Bobkova et al., 2015, 2014). Briefly, human Hsp 70 containing six Histidine amino acids was expressed in *E. coli* or army worm cells through the Bac‐to‐Bac system using the plasmid pFastBacHTb‐Hsp70 driven by the polyhedron promoter. RHsp70 was isolated from the cells through the Ni‐NTA resin columns, and its purity was determined by Coomassie Blue staining and Western blot using the 3B5 anti‐Hsp70 and N69 anti‐Hsc70 antibodies. The Bradford's protocol was followed to measure the concentration of rHsp70. All animal procedures were approved by Longyan People Hospital of Fujian (d78.j2).

Eighteen‐month‐old male mice (a total number of 162) were purchased from Shanghai Model Organisms (Shanghai, China) and housed in an environmentally controlled facility (12 h light/12 h dark cycle, room temperature of 22–24°C, 60% humidity). The previous investigation also testified that 2.5% sevoflurane (Yang et al., 2020) and 3%–7% sevoflurane (Xu & Qian, 2020) could be utilized to induce anesthesia in the chamber. In this study, three different concentrations of sevoflurane (1.5%, 3%, and 7%) were used to anesthetize mice. Control mice were placed in the anesthesia box and were exposed to regular air for 3 h. A subset of mice was treated with rHsp70 (29.4, 58.8, and 117.6 μg/kg) through intranasal injections once per day for one week and were then exposed to 3% sevoflurane. Animals were anesthetized by intraperitoneal injection of a lethal dose of pentobarbital. The whole brain was rapidly removed and the hippocampus was dissected out for the biochemical assays.

### 2.2 Morris water maze test

Mouse cognitive function, including spatial learning and memory, was assessed through Morris water maze test as described previously (Yang et al., 2020). Mice were subjected to the behavioral assessment at 48 h following sevoflurane exposure. Briefly, opaque water was filled into a circular tank with a height of 60 cm and a diameter of 200 cm and was maintained at 25°C. A 10 cm diameter platform was placed 1 cm underneath the water surface in one quadrant. The test was consisted of 6 days, including 5 days of training period and 1 day of trial session. During the training, each mouse was placed into the tank and allowed a maximum of 60 s to explore and search for the platform. The mouse stayed on the platform for a total of 10 s. The mouse was directed to the platform if it was not found within 60 s. Escape latency and speed of swimming were measured. During the probe trial, the platform was removed, and the mouse was placed in the quadrant opposite to the quadrant of the platform previously (target quadrant). Each mouse was allocated 120 s to explore the pool. The duration in the target quadrant and the number of platform crossings were recorded.

### 2.3 Enzyme‐linked immunosorbent assay

Levels of Hsp70 in the serum and hippocampus were determined by enzyme‐linked immunosorbent assay (ELISA). The Hsp70 ELISA Kit (ab133060, Abcam) was used for assessing Hsp70 levels in the hippocampus. The Hsp70 High Sensitivity ELISA Kit (ab133061, Abcam) was used for assessing Hsp70 levels in mouse serum. The following kits were used for the detection of inflammatory cytokines in the hippocampus: Mouse IL‐6 ELISA kit (ab100713, Abcam) for IL‐6, Mouse IL‐1 beta ELISA Kit (ab197742) for IL‐1ß, and Mouse monocyte chemoattractant protein‐1 (MCP1) ELISA Kit (ab100722) for MCP1. ELISA was performed according to the manufacturer's instruction.

### 2.4 Quantitative reverse transcription polymerase chain reaction

Hippocampal mRNA levels of relevant genes were assessed by quantitative reverse transcription polymerase chain reaction (RT‐qPCR). cDNA was synthesized by reverse transcription using the total RNA isolated from the hippocampus. To evaluate the expression levels of each target gene, 2‐^△△^Ct values were then calculated. The mRNA expressions of each target genes were normalized to the β‐actin.

### 2.5 Western blot

Mice were euthanized, and hippocampus was isolated and homogenized. To determine NF‐κB activation, cytoplasmic and nuclear proteins were prepared with a nuclear and cytoplasmic protein extraction kit according to a previous study (Liu et al., 2020). An equivalent number of proteins from total hippocampus lysate or cytoplasmic and nuclear extractions were subjected to protein gel electrophoresis. Relevant proteins were detected after incubation with primary and secondary antibodies. The following primary antibodies were used in this study: anti‐Hsp70 (1:1000, Cell Signaling Technology), anti‐GAPDH (1:2000, Sigma), anti‐Bax (1:800, Abcam), anti‐Bcl‐2 (1:1000, Abcam), anti‐NF‐κB p65 (1:500, Abcam), and anti‐phospho(p)‐p65 (1:1000, Abcam).

### 2.6 Statistical analysis

Data were analyzed by SPSS software. Two‐way analysis of variance (ANOVA) followed by Tukey's multiple comparisons test was performed for comparisons of different groups in the training sessions of Morris water maze test. One‐way ANOVA followed by Dunn's multiple comparisons test was used to determine the statistical differences among different groups. Data were represented by mean ± SD. Differences with *p* < .05 were considered statistically significant.

## 3 RESULTS

### 3.1 Sevoflurane anesthesia impairs cognitive function and suppresses Hsp70 production in aged mice

**FIGURE 1:**
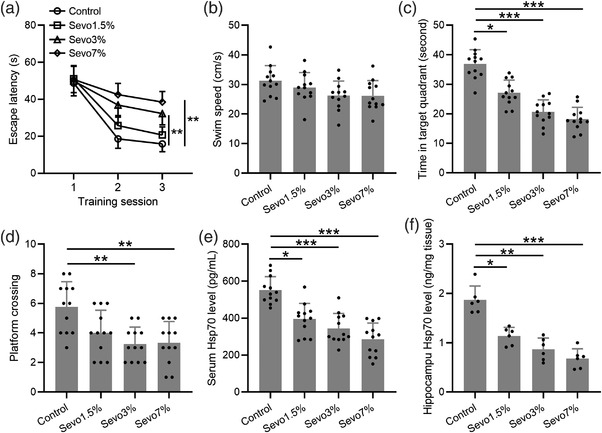
Sevoflurane exposure induced cognitive impairment and suppressed Hsp70 protein levels in aged mice. (a–d) Spatial learning and memory of aged mice were determined by the Morris water maze. Escape latencies (a) and average speed of swimming (b) were recorded in the three training sessions. The duration in the target quadrant (c) and the number of times crossing the platform site during the probe trial were recorded. The Hsp70 concentrations in plasma (e) and (f) were determined in mice at 48 h after sevoflurane anesthesia with different concentrations for 3 h. Twelve mice were used for each group. Data were represented by mean ± SD. *p < .05, **p < .01, ***p < .001

We first confirmed the effects of sevoflurane on the cognitive function of aged mice by Morris water maze test. To do that, we first exposed aged mice to different concentrations of sevoflurane for 3 h and carried out the behavioral assay at 48 h following anesthesia. We found that sevoflurane dose‐dependently impaired learning and memory in aged mice. During the training sessions, mice exposed to 3% and 7% sevoflurane showed significantly increased escape latency during the third training session (Figure 1a), with the average swim speed not impacted (Figure 1b). During the probe trial, mice spent significantly less time in the target quadrant after being exposed to sevoflurane at all three concentrations examined (1.5%, 3%, and 7%) (Figure 1c), and sevoflurane also dose‐dependently reduced the number of platform crossing (Figure 1d).

We then examined the impact of sevoflurane on the levels of Hsp70 in the serum and hippocampus of aged mice and found that sevoflurane at all the concentrations examined significantly suppressed Hsp70 production both in the serum (Figure 1e) and hippocampus (Figure 1f).

### 3.2 Exogenous rHsp70 restores Hsp70 levels following sevoflurane anesthesia in aged mice

**FIGURE 2:**
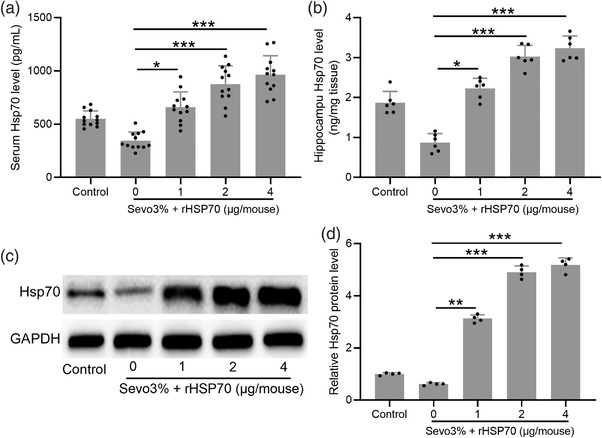
Exogenous rHsp70 restored Hsp70 expressions in aged mice under sevoflurane anesthesia. Enzyme‐linked immunosorbent assay (ELISA) was used to measure the Hsp70 levels in serum (a) and hippocampus (b). Quantitative reverse transcription polymerase chain reaction (RT‐qPCR) was used to measure the mRNA expression of Hsp70 in the hippocampus (c). Hippocampal Hsp70 protein expression was determined by Western blot analysis (d). Twelve mice were used for each group. For the experiments of RT‐qPCR and Western blotting, the hippocampal homogenate from 12 mice in each group was mixed, and the experiments were repeated for four times. Data were represented by mean ± SD. *p < .05, **p < .01, ***p < .001

To determine the impact of exogenous rHsp70 on sevoflurane‐induced neurotoxicity, we first examined whether pretreatment of mice with rHsp70 for 1 week prior to sevoflurane exposure could restore Hsp70 levels. ELISA analysis showed that rHsp70 dose‐dependently prevented the reduction of Hsp70 in the serum (Figure 2a) and hippocampus (Figure 2b). Finally, an examination of Hsp70 protein expression in the hippocampus showed that sevoflurane suppressed Hsp70 protein expression, which was significantly reversed by exogenous rHsp70 (Figure 2c,d).

### 3.3 Exogenous rHsp70 prevents cognitive impairment following sevoflurane anesthesia in aged mice

**FIGURE 3:**
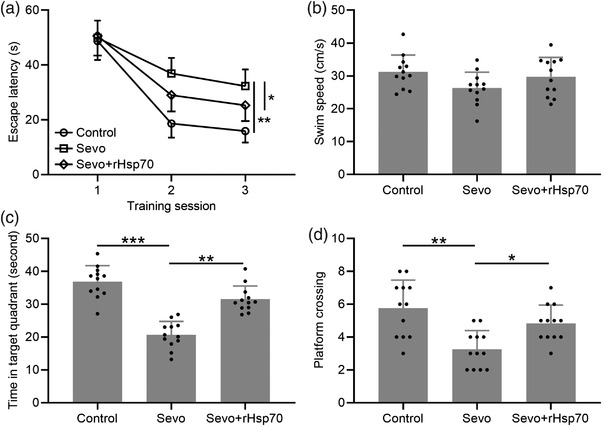
Impact of exogenous rHsp70 on the cognitive injuries induced by sevoflurane anesthesia in aged mice. Escape latencies (a) and average speed of swimming (b) were recorded in the three training sessions. The duration in the target quadrant (c) and the number of times crossing the platform site during the probe trial were recorded. Twelve mice were used for each group. Data were represented by mean ± SD. *p < .05, **p < .01, ***p < .001

We then assessed whether exogenous rHsp70 could suppress sevoflurane‐induced disruption in spatial learning and memory in the Morris water maze test. In training sessions, we found that mice pretreated with rHsp70 showed improved spatial learning ability after exposure to sevoflurane, although the escape latency was still significantly longer than that of control mice (Figure 3a). We showed that the improvement in spatial learning was not due to increased swim ability since rHsp70 did not significantly alter the swim speed of mice after exposure to sevoflurane (Figure 3b). During the probe test, mice exposed to sevoflurane spent significantly less time in the target quadrant as expected, and pretreatment of mice prior to sevoflurane exposure significantly increased the time in the target quadrant (Figure 3c). Consistently, rHsp70 restored the number of platforms crossing of mice after exposing them to sevoflurane (Figure 3d). These results suggested that exogenous rHsp70 significantly prevented sevoflurane anesthesia‐induced cognitive impairment.

### 3.4 Exogenous rHsp70 suppresses sevoflurane‐induced neurotoxicity in aged mice

**FIGURE 4:**
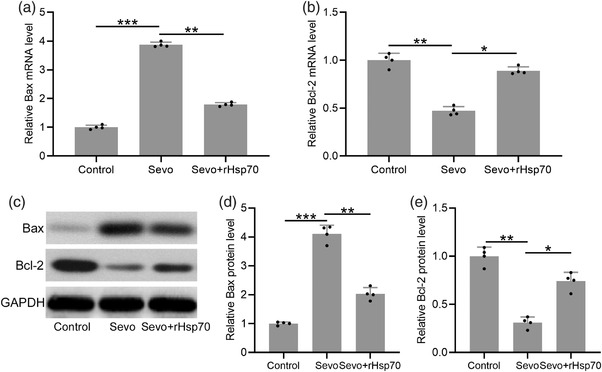
Impact of exogenous rHsp70 on the cell apoptosis in the hippocampus induced by sevoflurane anesthesia in aged mice. Hippocampal mRNA levels of Bax (a) and Bcl‐2 (b) were determined by quantitative reverse transcription polymerase chain reaction (RT‐qPCR). Bax and Bcl‐2 protein expression was assessed by Western blot (c) and quantified (d and e). Data were represented by mean ± SD. Twelve mice were used for each group. For the experiments of RT‐qPCR and Western blotting, the hippocampal homogenate from 12 mice in each group was mixed, and the experiments were repeated for four times. *p < .05, **p < .01, ***p < .001

To determine whether exogenous rHsp70 could suppress sevoflurane‐induced neurotoxicity, we examined the levels of pro‐apoptotic Bax and anti‐apoptotic Bcl‐2. Consistently with induction of neurotoxicity by sevoflurane, we found that sevoflurane exposure resulted in a significant increase in the mRNA level of Bax (Figure 4a) and a significant reduction in Bcl‐2 mRNA expression (Figure 4b). Similarly, Western blot analysis (Figure 4c) showed that sevoflurane significantly increased Bax (Figure 4d) and reduced Bcl‐2 protein expression (Figure 4e). Consistent with the role of rHsp70 in suppressing sevoflurane‐induced neurotoxicity, we found that rHsp70 significantly suppressed Bax and enhanced Bcl‐2 expression in mice exposed to sevoflurane.

### 3.5 Exogenous rHsp70 prevents sevoflurane‐induced neuroinflammation in aged mice

**FIGURE 5:**
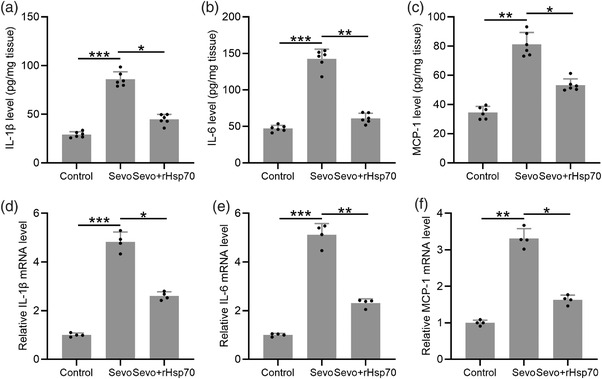
Effects of exogenous rHsp70 on the inflammatory responses in the hippocampus induced by sevoflurane anesthesia in aged mice. Interleukin‐1 beta (IL‐1β) (a), interleukin 6 (IL‐6) (b), and monocyte chemoattractant protein‐1 (MCP‐1) (c) concentrations in the hippocampus were assessed using enzyme‐linked immunosorbent assay (ELISA). IL‐1β (d), IL‐6 (e), and MCP‐1 (f) mRNA expression in the hippocampus were tested through quantitative reverse transcription polymerase chain reaction (RT‐qPCR). Six mice were used for each group. Data were represented by mean ± SD. *p < .05, **p < .01, ***p < .001

To confirm the findings in previous studies, we examined the levels of proinflammatory cytokines in the hippocampus following sevoflurane exposure. ELISA analysis showed that the concentrations of interleukin‐1 beta (IL‐1ß) (Figure 5a), interleukin‐6 (IL‐6) (Figure 5b), and MCP‐1 (Figure 5c) were significantly increased in the hippocampus of mice exposed to sevoflurane. Consistently, we found that the mRNA expressions of IL‐1ß (Figure 5d), IL‐6 (Figure 5e), and MCP‐1 (Figure 5f) in the hippocampus were also significantly induced by sevoflurane exposure. Importantly, we found that mice pretreated with rHsp70 prior to sevoflurane exposure significantly restored the concentration of the proinflammatory cytokines as well as their mRNA expression in the hippocampus.

### 3.6 Exogenous rHsp70 suppressed sevoflurane‐induced NF‐κB activation in aged mice

**FIGURE 6:**
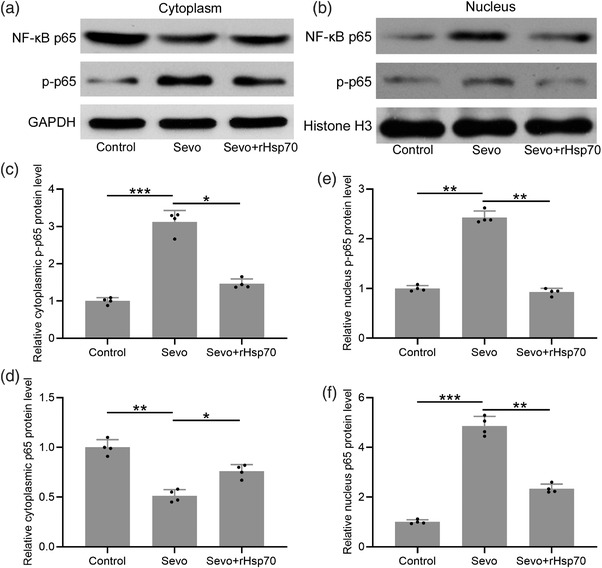
Impact of exogenous rHsp70 on NF‐κB activation in the hippocampus of aged mice under sevoflurane anesthesia. Cytosolic (a) and nuclear (b) p‐p65 and p65 levels were assessed by Western blot. The quantifications were shown in (c) to (f). Twelve mice were used for each group. The hippocampal homogenate from 12 mice in each group was mixed, and the experiments were repeated for four times. Data were represented by mean ± SD. *p < .05, **p < .01, ***p < .001

Finally, to investigate the molecular mechanism of rHsp70‐mediated suppression of sevoflurane‐induced proinflammatory cytokine production, we examined the NF‐κB signaling by Western blot analysis of the NF‐κB p65 subunit in the cytoplasm (Figure 6a) and nucleus (Figure 6b) and its phosphorylation status. As anticipated, we found that sevoflurane anesthesia significantly increased the levels of p‐p65 both in the cytoplasm (Figure 6c) and nucleus (Figure 6e). Sevoflurane exposure leads to nucleus translocation of p65, leading to significantly reduced cytoplasmic p65 protein level (Figure 6d) and increased nucleus portion (Figure 6f) compared to that of the control treatment. Importantly, we found that sevoflurane‐induced p65 nucleus translocation and phosphorylation were suppressed by pretreatment of the mice with exogenous rHsp70. Our results suggest that sevoflurane‐induced NF‐κB activation is prevented by exogenous rHsp70 prior to induction of anesthesia in aged mice.

## 4 DISCUSSION

In this study, we investigate whether exogenous rHsp70 could prevent cognitive impairment in aged mice resulting from sevoflurane exposure and determine the neuroprotective role of rHsp70. We show here that aged mice exposed to sevoflurane have cognitive decline and reduced Hsp70 levels in the serum and hippocampus following sevoflurane exposure. RHsp70 pretreatment prior to sevoflurane exposure restores Hsp70 levels in the serum and hippocampus and prevents sevoflurane‐induced disruption in learning and memory. Our results reveal a neuroprotective effect of rHSP70 in response to sevoflurane anesthesia. RHsp70 substantially suppresses pro‐apoptotic Bax and increases anti‐apoptotic Bcl‐2. Additionally, rHsp70 significantly inhibits sevoflurane‐induced pro‐inflammatory cytokines production. Finally, our study reveals that rHsp70 suppresses sevoflurane‐induced neuroinflammation through NF‐κB signaling. Our study, for the first time, shows that exogenous rHsp70 is neuroprotective and suppresses cognitive decline in response to sevoflurane stimulation. This finding is of great clinical implication for the potential of preventing POCD in elderly patients by targeting Hsp70.

Sevoflurane is a frequently used inhalable anesthetic for induction of anesthesia during surgery. Unfortunately, sevoflurane is associated with POCD and has been shown to lead to delayed cognitive functional recovery among elderly patients undergoing major cancer surgery (Zhang et al., 2018). Cognitive decline is frequently observed in animals exposed to sevoflurane in young and aged rodents. Our study confirms that the aged mice exhibit impaired learning and memory function through behavior assessment upon exposure to sevoflurane. Interestingly, we also observe a significant reduction of Hsp70 concentrations in the serum and hippocampus of the aged mice due to sevoflurane exposure. Although a previous study by Liu et al. (2020) shows a slight upregulation of Hsp70 protein expression in the hippocampus of rats by exposure to sevoflurane, other studies report the opposite trend. Consistent with our study, F. Yu et al. (2020) show that sevoflurane suppresses Hsp70 expression in a rat model of cerebral ischemia/reperfusion injury.

The role of Hsp70 in neuroprotection has been established previously. In the absence of sevoflurane exposure, exogenous rHsp70 has been shown to improve cognition and prolong the life span of aging mice (Bobkova et al., 2015) and reduce the accumulation of toxic amyloid beta plaque and improve memory in Alzheimer's disease mouse models (Bobkova et al., 2014). Our study consistently shows that intranasal administration of exogenous rHsp70 restores Hsp70 levels in response to sevoflurane exposure and significantly alleviates sevoflurane‐induced cognitive impairment as manifested by improved spatial learning and memory ability.

In search for the mechanism through which exogenous rHsp70 exerts its neuroprotective role, we focus on two aspects of sevoflurane‐induced neurotoxicity, including neuronal survival and neuroinflammation. It has been previously shown that isoflurane inhalation induces caspase‐3 activation, a marker for apoptosis, in mice brains (Xie et al., 2008). A plethora of studies have also showed that sevoflurane induces neuronal apoptosis in different cellular and animal models, including neonatal and aged rodents (Huang et al., 2018; Liu et al., 2020; Sun et al., 2015). In our study, we also examine markers of apoptosis in the hippocampus of aged mice following sevoflurane anesthesia. Consistent with previous studies, we show significant induction of Bax expression and suppression of Bcl‐2 expression after sevoflurane exposure, which corresponds to sevoflurane‐induced neuronal apoptosis. Importantly, our study reveals a significant protective effect of exogenous rHsp70 against sevoflurane‐induced apoptosis in the hippocampus, as evidenced by suppressed Bax and enhanced Bcl‐2 expression in mice pretreated with rHsp70, prior to sevoflurane exposure. A previous study shows that Hsp70 is required to prevent sevoflurane‐induced apoptosis (Liu et al., 2020). Taken together, our findings, as well as others, suggest that Hsp70 plays an important role in preventing sevoflurane‐induced neuronal loss.

In addition to neuronal loss, sevoflurane has also been shown to induce neuroinflammation, leading to increased proinflammatory cytokines production and NF‐κB signaling activation (Gao et al., 2020; Liu et al., 2020). Consistently, our study also detects significantly enhanced pro‐inflammatory cytokines production induced by sevoflurane exposure in the hippocampus of aged mice. Importantly, we show that exogenous rHsp70 efficiently blocks sevoflurane‐induced pro‐inflammatory cytokine production. Further analysis reveals that the NF‐κB signaling is suppressed by rHsp70 treatment, which corresponds to dampened neuroinflammation in mice exposed to sevoflurane. Our finding is consistent with the study by Liu et al. (2020), which also shows that Hsp70 is required to suppress sevoflurane‐induced neuroinflammation. On the other hand, other studies have also revealed the role of Hsp70 in suppressing neuroinflammation induced by α‐synuclein stimulations (Kim et al., 2012; W. W. Yu et al., 2018) and NF‐κB activation (H. Li, Yang, et al., 2019).

## 5 CONCLUSION

In summary, exogenous rHsp70 effectively blocks sevoflurane‐induced neurotoxicity and cognitive impairment, which indicates the possibility of targeting Hsp70 to prevent POCD in elderly patients. Our important findings warrant further investigation of the clinical application of rHsp70 in combination with anesthetics before surgery.

## CONFLICT OF INTEREST

The authors declare no conflict of interest.

### PEER REVIEW

The peer review history for this article is available at https://publons.com/publon/10.1002/brb3.2861

## Data Availability

Data could be obtained upon reasonable request to the corresponding author.
